# Differential cellular localization of antioxidant enzymes in the trigeminal ganglion

**DOI:** 10.1186/1129-2377-14-S1-P83

**Published:** 2013-02-21

**Authors:** M Shibata, H Sato, T Shimizu, S Shibata, H Toriumi, T Kuroi, T Ebine, T Iwashita, M Funakubo, C Akazawa, K Wajima, T Nakagawa, H Okano, N Suzuki

**Affiliations:** 1 Department of Neurology, School of Medicine, Keio University, Japan; 2 Department of Physiology, School of Medicine, Keio University, Japan; 3 Department of Biochemistry and Biophysics, Graduate School of Health and Sciences, Tokyo Medical and Dental University, Japan; 4 Department of Dentistry and Oral Surgery, School of Medicine, Keio University, Japan; 5 Department of Neurology, School of Medicine, Keio University, UK

## Background

Because of its high oxygen demands, neural tissue is predisposed to oxidative stress. In the present study, we aimed to clarify the cellular localization of antioxidant enzymes in the trigeminal ganglion. The transient receptor potential vanilloid subfamily member 1 (TRPV1) is implicated in inflammatory hyperalgesia. We also explored the effect of TRPV1 stimulation on the production of reactive oxygen species (ROS).

## Methods and results

We used 14 adult transgenic mice expressing the efficient fluorescent protein, Venus, under the control of the Sox10 promoter. TG sections were prepared for immunostaining. The colocalization of Venus signal with glutamate/aspartate transporter, a marker for satellite glial cells (SGCs), was observed. Whereas both superoxide dismutases (SODs) 1 and 2 were present in neurons, only SOD 1 was identified in SGCs. The enzymes relevant to hydrogen peroxide degradation displayed differential cellular localization, such that neurons were endowed with glutathione peroxidase 1 and thioredoxin 2, and catalase and thioredoxin 2 were present in SGCs. Moreover, only SGCs were labeled by the oxidative damage marker, 8-hydroxy-2'-deoxyguanosine, which indicates that the antioxidant systems of SGCs were less potent. We established PC12 stable transformants expressing enhanced green fluorescent protein (EGFP)-full-length TRPV1 fusion protein. It was found that TRPV1 agonist stimulation in the presence of TRPV1 overexpression caused a robust increase in the reactive oxygen species and in caspase-3 activation. Caspase-3 activation was inhibited by the reactive oxygen species scavenger, TEMPOL.

## Conclusion

This study delineates the localization of antioxidative stress-related enzymes in the trigeminal ganglion and reveals the importance of the pivotal role of the reactive oxygen species in TRPV1-mediated caspase-3 activation. Therapeutic measures for antioxidative stress should be taken to prevent damage to trigeminal primary sensory neurons in inflammatory pain disorders, such as meningitis and possibly migraine.

